# CRNDE enhances the expression of MCM5 and proliferation in acute myeloid leukemia KG-1a cells by sponging miR-136-5p

**DOI:** 10.1038/s41598-021-96156-3

**Published:** 2021-08-18

**Authors:** Chen Liu, Liang Zhong, Chenlan Shen, Xuan Chu, Xu Luo, Lihua Yu, Jiao Ye, Ling Xiong, Wenran Dan, Jian Li, Beizhong Liu

**Affiliations:** 1 Central Laboratory of Yong-Chuan Hospital, Chongqing Medical University, Chongqing, 402160 China; 2 Key Laboratory of Laboratory Medical Diagnostics, Ministry of Education, Department of Laboratory Medicine, Chongqing Medical University, Chongqing, 400016 China; 3 Clinical Laboratory of Yong-Chuan Hospital, Chongqing Medical University, Chongqing, 402160 China

**Keywords:** Cancer, Cell biology, Molecular biology

## Abstract

The long-noncoding RNA colorectal neoplasia differentially expressed (CRNDE) gene has been considered to be crucial in tumor malignancy. Although CRNDE is highly expressed in acute myeloid leukemia (AML), its mechanism of action remains unknown. In this study, GEPIA and qRT-PCR were performed to confirm the expression of CRNDE in AML samples and cell lines, respectively. CRNDE shRNA vectors were transfected to explore the biological functions of CRNDE. The cell proliferation was assessed by the CCK8 assay, while apoptosis and cell cycle distribution were measured by flow cytometry and Western blotting. The results showed that CRNDE was overexpressed in both AML samples and cell lines. CRNDE silencing inhibited proliferation and increased apoptotic rate and cell cycle arrest of KG-1a cells. The luciferase reporter assay coupled with RIP assay revealed that CRNDE act as a ceRNA. Rescue assays demonstrated that the effects of CRNDE silencing could be reversed by miR-136-5p inhibitors. In conclusion, our results expound that the CRNDE/miR-136-5p/MCM5 axis modulates cell progression and provide a new regulatory network of CRNDE in KG-1a cells.

## Introduction

Acute myeloid leukemia (AML) is a hematologic malignancy, characterized by aberrant proliferation and differentiation of haematopoietic stem cells (HSCs)^1^. Compared to other types of leukemia, AML is relatively common in adults and its prevalence increases with age^2,3^.

Long non-coding RNAs (lncRNAs) belong to the non-coding RNAs family^4^. LncRNAs work in several ways, such as regulating transcription in cis or trans, organizing nuclear domains, and regulating proteins or RNA molecules^5,6^. Recent investigations indicated that lncRNAs played a key role in the occurrence and development of tumor^7^. For example, Sun et al. revealed that the lncRNA ANRIL may promote malignant cell growth and glucose metabolism by regulating the ANRIL-AdipoR1-AMPK/SIRT1 signaling pathway^8^. Dong et al. showed that the lncRNA HOXA-AS2 regulates the chemoresistance of AML cells by competing for miR-520c-3p binding and through regulating S100A4 expression by acting as a ceRNA^9^.

The colorectal neoplasia differentially expressed (CRNDE) is a gene located on chromosome 16. It was first found to be overexpressed in colorectal adenomas and carcinomas and confirmed to play a critical role in many types of cancers, subsequently^10–12^. A recent study showed that CRNDE was up-regulated in AML patients’ bone marrow tissues and negatively correlated with patients’ overall survival^13^. However, the role of CRNDE in AML still needs further elucidation.

MicroRNAs (miRNAs) participate in various biological processes. Studies have found that miR-136-5p can regulate the occurrence and development of thyroid cancer by targeting mataderin (MTDH)^14^. However, up to now, the association between miR-136-5p and AML has not been reported in relevant studies.

In the present study, we demonstrate that CRNDE modulates KG-1a cells progression via the CRNDE-miR-136-5p-MCM5 axis, which provides a new regulatory network of CRNDE in KG-1a cells.

## Materials and methods

### Cell culture

The acute myeloid leukemia cell lines KG-1a, NB4 and HL60, and the human embryonic kidney 293T cell line were stored at our laboratory. RPMI-1640 medium (Gibco, USA), supplemented with 10% fetal bovine serum (FBS, USA) and 1% penicillin–streptomycin (Beyotime, China) was used to culture the AML cells. The 293T cells were cultured in Dulbecco’s Modified Eagle’s Medium (DMEM, Gibco, USA), supplemented with 10% fetal bovine serum (FBS, USA) and 1% penicillin–streptomycin (Beyotime Biotechnology, Shanghai, China). The cells were maintained at 37 °C with 5% CO_2_. The leukocytes were obtained from healthy human peripheral blood.

### Extraction of leukocytes in human peripheral blood

Fresh anticoagulants from healthy people were collected and lysed by red blood cell lysis buffer (Beyotime Biotechnology, Shanghai, China) for several times until the supernatant became clear and transparent. The remaining cells were washed twice with PBS. The precipitates obtained were human peripheral blood leukocytes, which were used as the control group of AML cell lines.

### Reverse transcription PCR and quantitative real-time PCR

TRIZOL reagent (Takara, Japan) was used to extract total RNA, that was transcribed into cDNA using the PrimeScript RT reagent Kit (Takara, Japan). Quantitative real-time PCR (qRT-PCR) was performed on a CFX Connect real-time PCR operating system (Bio-Rad, USA) using the SYBR Premix Ex Taq II (Takara, Japan) kit. Relative expression levels were calculated using the 2^−ΔΔCt^ method. U6 and β-actin were used as internal standards for miRNA and mRNA, respectively. The used primers are as follows: CRNDE(forward: 5ʹ-ATATTCAGCCGTTGGTCTTTGA-3ʹ, reverse: 5ʹ-TCTGCGTGACAACTGAGGATTT-3ʹ), miR-136-5p(forward: 5ʹ-CGCGACTCCATTTGTTTTGAT-3ʹ, reverse: 5ʹ-AGTGCAGGGTCCGAGGTATT-3ʹ, reverse transcription: 5ʹ-GTCGTATCCAGTGCAGGGTCCGAGGTATTCGCACTGGATACGACTCCATC-3ʹ), MCM5(forward: 5ʹ-AGCATTCGTAGCCTGAAGTCG-3ʹ, reverse: 5ʹ-CGGCACTGGATAGAGATGCG-3ʹ), β-Actin(forward: 5ʹ- TGACGTGGACATCCGCAAAG -3ʹ, reverse: 5ʹ-CTGGAAGGTGGACAGCGAGG -3’), U6(forward: 5’- GCTTCGGCAGCACATATACTAAAAT-3’, reverse: 5ʹ-CGCTTCACGAATTTGCGTGTCAT-3’). Relative expression levels were calculated using the 2^−ΔΔCt^ method. U6 and β-actin were used as internal standards for miRNA and mRNA, respectively.

### Cell transfection and infection

MiR-136-5p mimic, miR-136-5p inhibitor, the mimic and the inhibitor negative controls were purchased from GenePharma (Shanghai, China). Briefly, 1 × 10^6^ cells per well were seeded in a 6-well plate and transfected using Lipofectamine 2000 (Invitrogen, USA), according to the manufacturer’s protocols.

To geerate CRNDE silenced stable cell lines, we used a lentiviral vector, which was designed and synthesized by GenePharma (Shanghai, China). The KG-1a cells were transfected following the manufacturer’s protocols, and after 72 h incubation, the cells were selected by adding 5 μg/mL puromycin (Abcam, MA, UK) for approximatively 2 weeks to generate the stable cell lines.

### Cell proliferation assay

Cell proliferation was detected using the Cell Counting Kit-8 (CCK8; HY-K0301), that was purchased from MedChemExpress (NJ, USA). The transfected cells were seeded into 96-well plates at a density of 2 × 10^3^/well, according to the manufacturer’s protocols, and 10 μl of CCK8 was added to each well at 0, 12, 24, 36, 48 h, followed by an additional 2 h incubation. The OD value at 450 nm was measured using a microplate reader.

### Cell counting

The transfected cells were seeded into 6-well plates and cultured at 37 °C with 5% CO_2_ and the numbers of cells were counted at 0, 24, 48, 72 and 96 h.

### Flow cytometry

To assess cell apoptosis, the transfected cells were collected, washed twice with cold phosphate-buffered saline (PBS), centrifuged at 1000 rpm for 5 min and resuspended at approximatively 1 × 10^6^ cells in 500 μl PBS. For cell cycle analysis, the cells were washed twice, resuspended in 100μL PBS and 500 μl of pre-chilled 75% ethanol was slowly added prior an overnight storage at 4 °C. Apoptosis and cell cycle were assessed by CytoFLEX flow cytometry (Beckman, USA), and the Cytexpert and Kaluza Analysis software were respectively used for data acquisition and analysis.

### Western blot

The cells were collected, washed 3 times with cold PBS and treated with cold RIPA lysis buffer containing a protease inhibitor cocktail (Beyotime Biotechnology, Shanghai, China). The BCA protein assay kit was used to detect the protein concentration, according to the manufacturer’s protocol (Beyotime Biotechnology, Shanghai, China). The extracted proteins were separated on SDS-PAGE gels and transferred to PVDF membranes (Millipore, MA, USA). The membranes were blocked with 5% skim milk for 2 h at room temperature and then the following primary antibodies were added:β-Actin (Boster Biological Technology, CA, USA), MCM5 (Abcam, MA, UK), Bcl-2 (Abcam, MA, UK), CyclinD1 (Wanleibio, Shenyang, China), CyclinA2 (Wanleibio, Shenyang, China) and p53 (Wanleibio, Shenyang, China). This step was followed by the addition of horseradish peroxidase-conjugated secondary antibodies (1:4,000; Biosharp, China) and the signals were detected using an ECL (enhanced chemiluminescence) kit (Millipore, USA).

### Luciferase reporter assays

The luciferase reporter plasmids CRNDE-Wt, CRNDE-Mut, MCM5-Wt and MCM5-Mut were purchased from GeneCreate (Wuhan, China). The sequences were respectively cloned into a pmirGLO Dual-luciferase plasmid to construct the luciferase reporter vectors. Lipofectamine 2000 (Invitrogen, CA, USA) was used to co-transfect the luciferase reporter plasmids and miR-136-5p mimic or mimic negative control into 293T cells. After 48 h culture, the transfected cells were collected and the luciferase activity measured using the Dual-Luciferase Reporter Assay System kit (Promega, WI, USA). The Firefly luciferase activity was standardized by the Renilla luciferase.

### RNA immunoprecipitation (RIP) assay

An RNA Binding Protein Immunoprecipitation kit (Millipore, Burlington, MA) was used to determine whether CRNDE exists in the miRNA mediated RISC complex. The cells were lysed in lysis buffer and anti-Ago2 antibody or anti-IgG magnetic beads were used to immunoprecipitate target RNA binding proteins and then the precipitated RNA was separated. QRT-PCR was performed to analyze the expression of CRNDE in the RNA obtained.

### Statistical analysis

Each experiment included at least 3 independent replicates and all values were shown as means ± SD. All statistics were analyzed using GraphPad Prism 6. The comparison between the groups was analyzed using the Student’s t-test or the one-way analysis of variance (ANOVA). A *p *value < 0.05 was considered significant (*) and a *p *value < 0.01 was considered as highly significant (**).

### Ethics approval and consent to participate

All experimental protocols were approved by Chongqing Medical University. Protocols involving human research participants had been performed in accordance with the Declaration of Helsinki and informed consent was obtained from all subjects.

## Results

### CRNDE is highly expressed in AML

**Figure 1:**
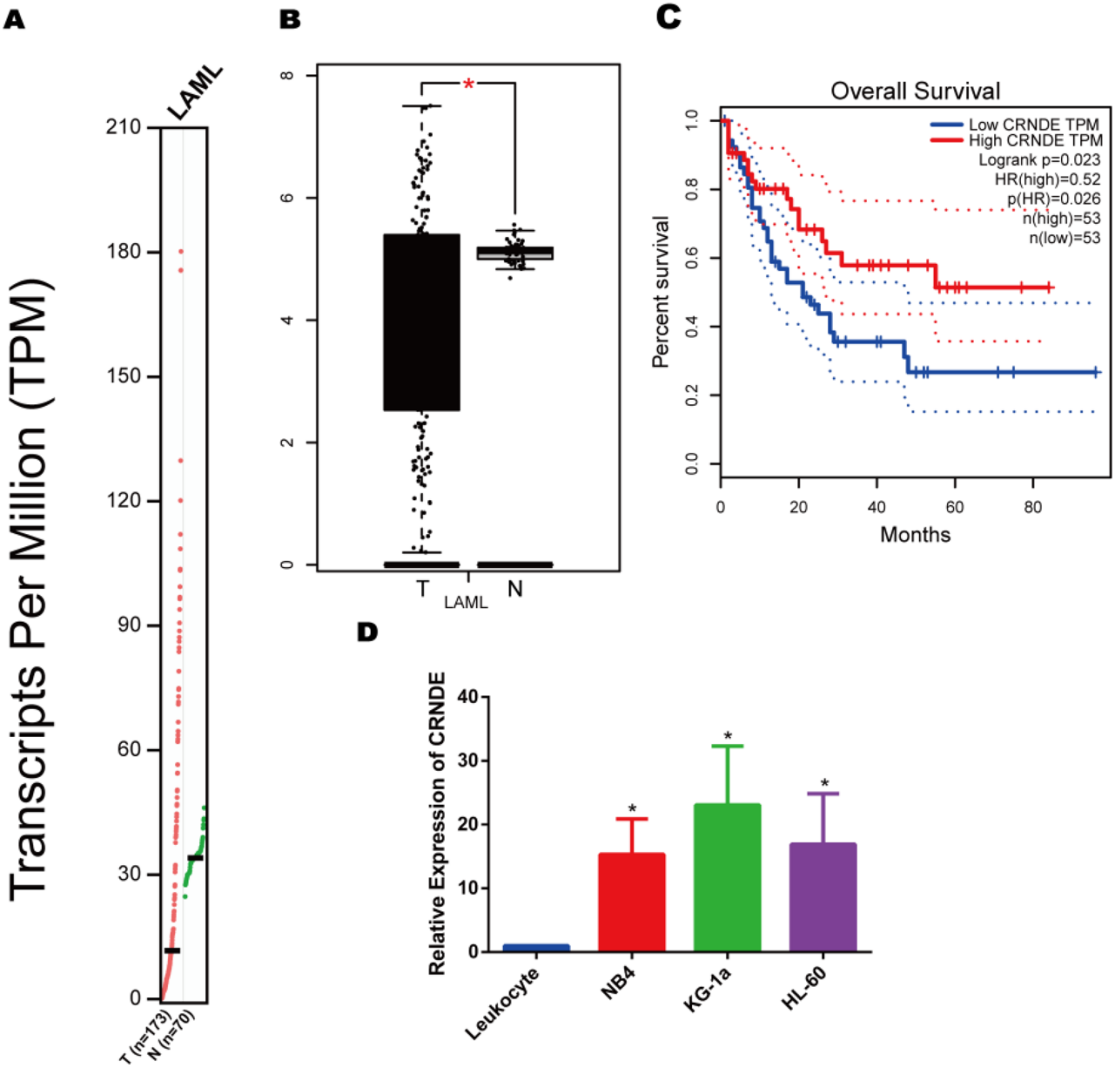
High expression of CRNDE in AML. (**a**,**b**) The expression of CRNDE in normal samples (n = 70) and AML samples (n = 173) from GEPIA (http://gepia.cancer-pku.cn/index.html) analysis. (**c**) The overall survival curves of CRNDE in AML patients from GEPIA analysis. (**d**) CRNDE expression in AML cell lines was detected by qRT-PCR. *p < 0.05, **p < 0.01.

To investigate the expression of lncRNA CRNDE in AML, we first analyzed the expression of CRNDE in The Cancer Genome Atlas (TCGA) database by GEPIA. The results showed that CRNDE was significantly up-regulated in AML samples (n = 173), compared with that in normal samples (n = 70) (Fig. 1a,b). However, to our surprise, CRNDE high expression correlated with AML better overall survival (OS) (Fig. 1c). Next, we measured the CRNDE expression in AML cell lines by qRT-PCR and the results showed that CRNDE was significantly overexpressed in AML cell lines (KG-1a, NB4 and HL60) compared with leukocytes (Fig. 1d).

### CRNDE knockdown inhibits proliferation and promotes apoptosis in KG-1a cells

**Figure 2:**
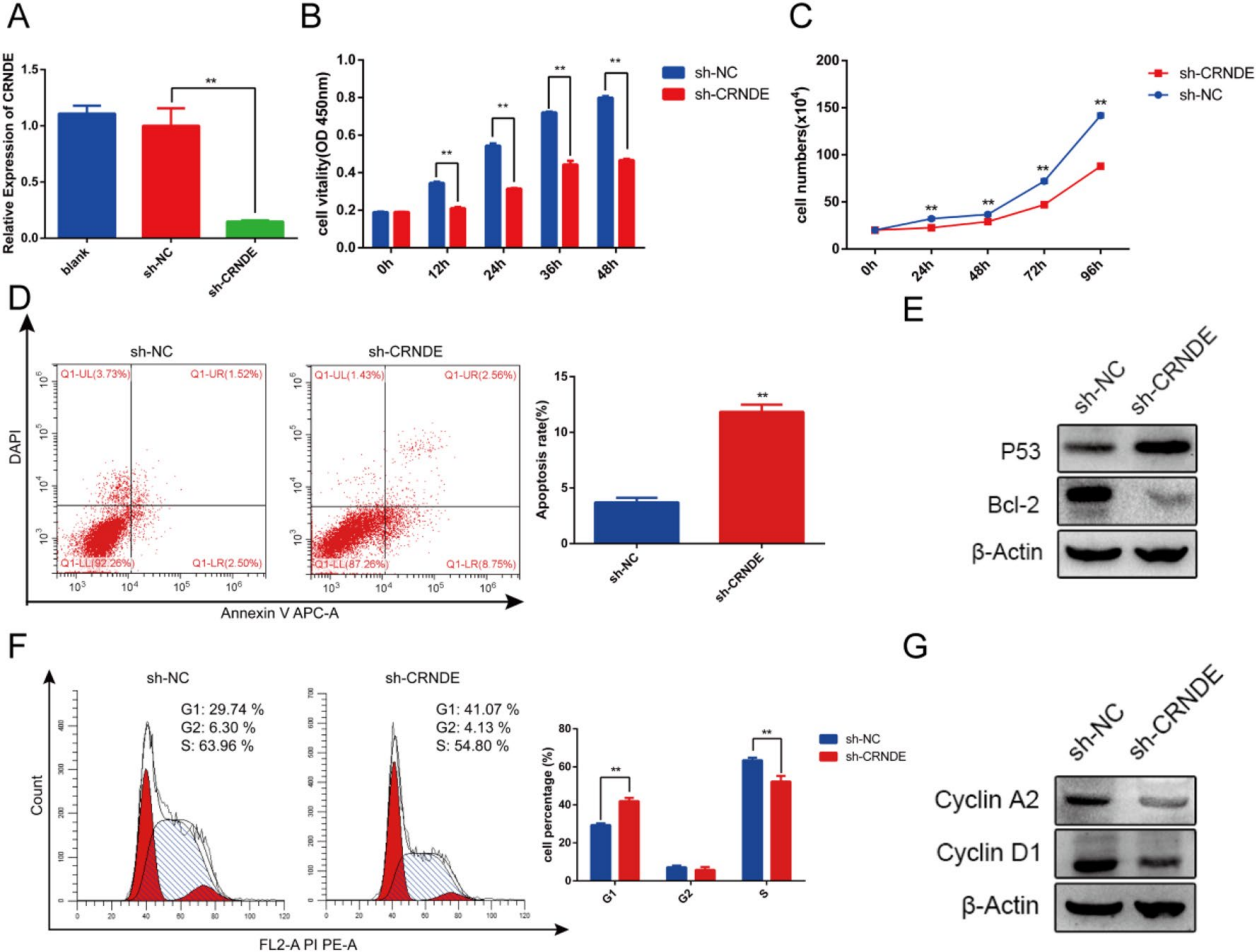
The effects of CRNDE knockdown on KG-1a cells proliferation, apoptosis and cell cycle distribution. (**a**) The transfection efficiency was verified by qRT-PCR. (**b**,**c**) Cell viability was analyzed by the CCK8 assay and cell counting assay. (**d**,**e**) Cell apoptosis was detected by flow cytometry and western blot. (**f**,**g**) Cell cycle was detected by flow cytometry and western blot. *p < 0.05, **p < 0.01.

To further explore the function of CRNDE, we transfected shRNAs targeting CRNDE (sh-CRNDE) into KG-1a cells to knock down CRNDE. The efficiency was assessed by qRT-PCR (Fig. 2a). The CCK8 and cell counting assay showed a significant inhibition of cell proliferation following CRNDE knockdown (Fig. 2b,c). Additionally, flow cytometry assays indicated that apoptosis was increased, and that the cell cycle was arrested at the G1 phase (Fig. 2d,f). The same results were obtained with Western blotting (Fig. 2e,g). In conclusion, CRNDE knockdown inhibits proliferation, promotes apoptosis, and blocks the cell cycle at G1 phase in KG-1a cells.

### CRNDE targets miR-136-5p and negatively regulates its expression

**Figure 3:**
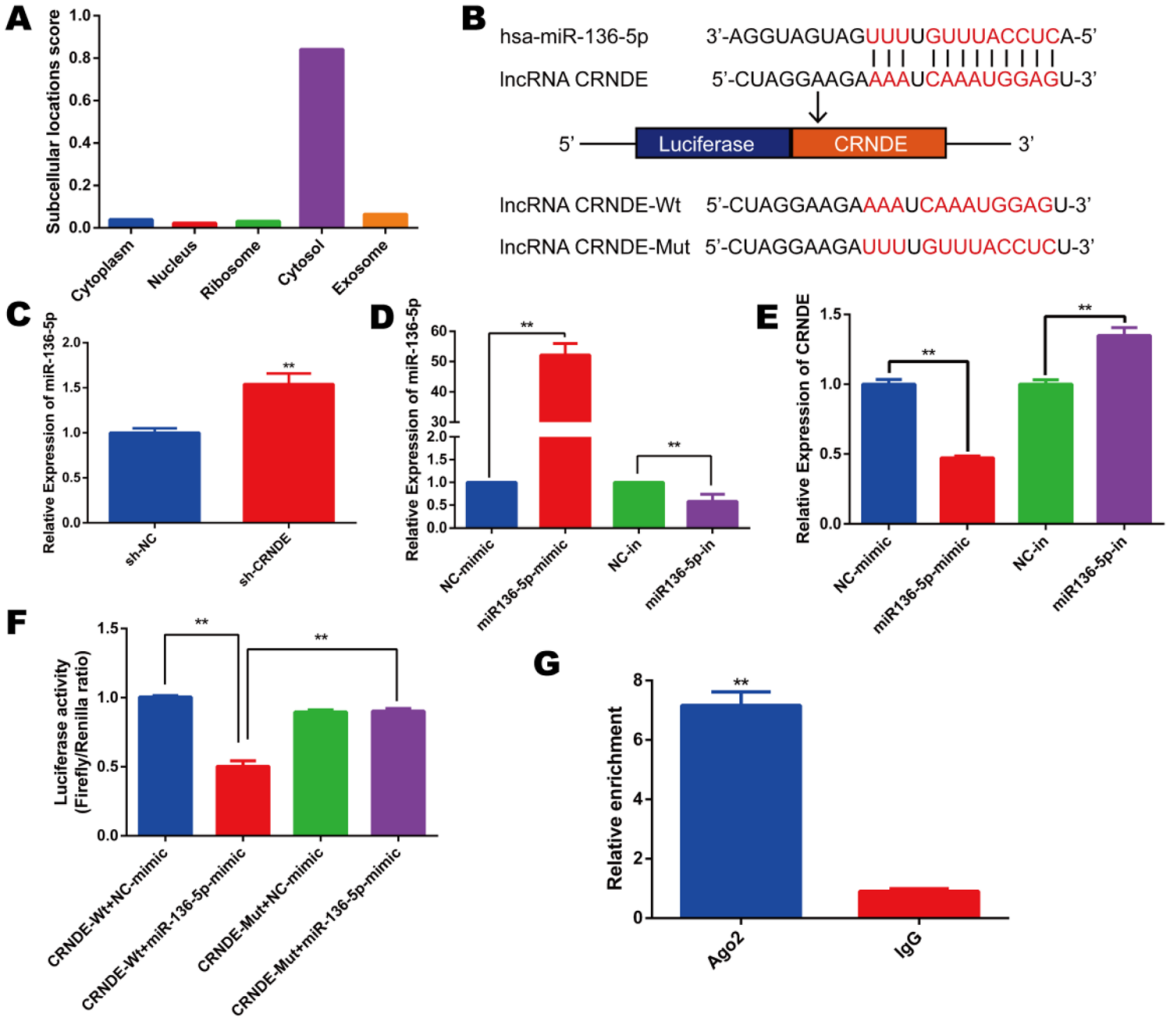
CRNDE binds to miR-136-5p. (**a**) The subcellular localization of CRNDE was predicted by lncLocator (http://www.csbio.sjtu.edu.cn/bioinf/lncLocator/). (**b**) The binding sites between CRNDE and miR-136-5p were predicted by Starbase V3.0 (http://starbase.sysu.edu.cn/) and LncBase V2.0 (http://carolina.imis.athena-innovation.gr/diana_tools/web/index.php?r=lncbasev2%2Findex-experimental). (**c**) QRT-PCR assay was used to detect the expression of miR-136-5p after knocking down CRNDE. (**d**) QRT-PCR assay was used to detect the transfection efficiency of miR-136-5p mimic and inhibitor. (**e**) QRT-PCR assay was used to determine the expression of CRNDE after transfecting miR-136-5p mimics and inhibitors. (**f**) Luciferase reporter assay for analysis of the interaction between miR-136-5p and CRNDE. (**g**) RIP assay was performed to estimate CRNDE expression in Ago2 immunoprecipitates. *p < 0.05, **p < 0.01.

The subcellular localization of lncRNA is closely related to its potential mechanism. LncLocator^15^ predicted that CRNDE was mainly located in the cytosol, which reminded that CRNDE most likely acted as a competing endogenous RNA (ceRNA) (Fig. 3a). Next, the prediction software Starbase V3.0 and LncBase V2.0 were used to determine its targets and we found the presence of a putative interaction site between CRNDE and miR-136-5p (Fig. 3b). With the silencing of CRNDE, miR-136-5p expression was significantly boosted in KG-1a cells (Fig. 3c). Additionally, miR-136-5p specific mimics and inhibitors were transfected into KG-1a cells and qRT-PCR was used to detect the transfection efficiency (Fig. 3d). The results showed that miR-136-5p mimics inhibited CRNDE expression, while miR-136-5p inhibitors rescued its expression (Fig. 3e). Then, a luciferase reporter assay was performed according to the interaction site and its mutation. The results displayed a decline in luciferase activity in 293 T cells cotransfected with miR-136-5p-mimics and CRNDE-WT (Fig. 3f). Furthermore, RIP assay indicated that CRNDE was highly enriched in the anti-Ago2 group (Fig. 3g). Taken together, these results show that CRNDE binds to miR-136-5p.

### MiR-136-5p is downregulated in AML cell lines and acts as a tumor suppressor

**Figure 4:**
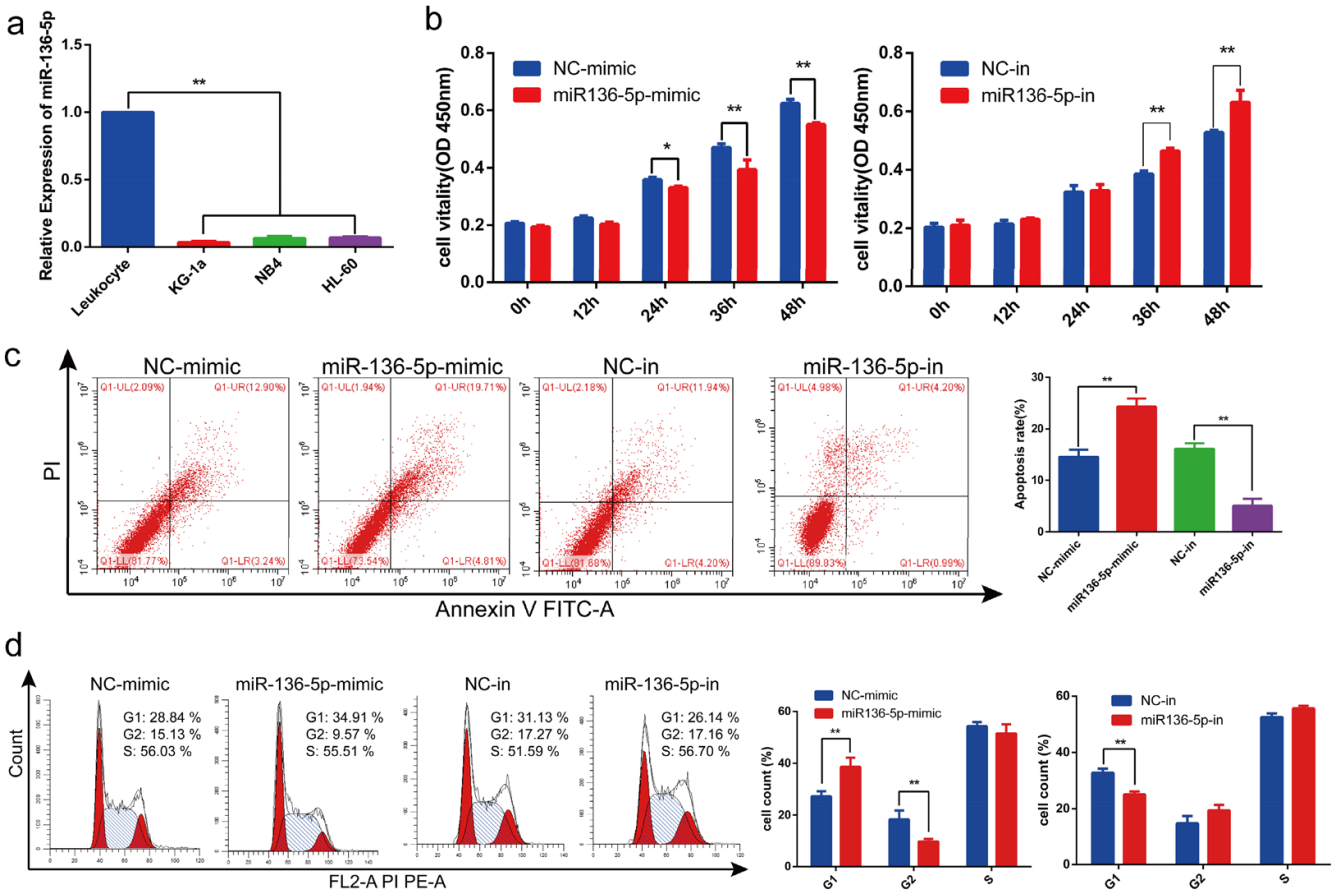
The effects of miR-136-5p on KG-1a cells proliferation, apoptosis and cell cycle distribution. (**a**) QRT-PCR was applied to determine the expression of miR-136-5p in AML cells. (**b**) Cell viability was analyzed by CCK‐8 assay. **c** Flow cytometry assay was performed to detect the apoptosis rate. (**d**) Flow cytometry assay was performed to detect cell cycle distribution. *p < 0.05, **p < 0.01.

QRT-PCR showed that miR-136-5p was significantly down-regulated in AML cell lines compared with leukocytes (Fig. 4a). The CCK8 assay revealed that cell proliferation was significantly suppressed by miR-136-5p mimics, while miR-136-5p inhibitors promoted proliferation in KG-1a cells (Fig. 4b). Furthermore, flow cytometry assays were performed to measure the apoptotic rate and cell cycle. The results showed the overexpression of miR-136-5p increased the apoptotic rate, whereas its down-regulation decreased it (Fig. 4c). And the cell cycle was blocked at G1 phase when miR-136-5p was overexpressed (Fig. 4d). These results demonstrate that miR-136-5p acts as a tumor suppressor.

### MCM5 is a direct target of miR-136-5p

**Figure 5:**
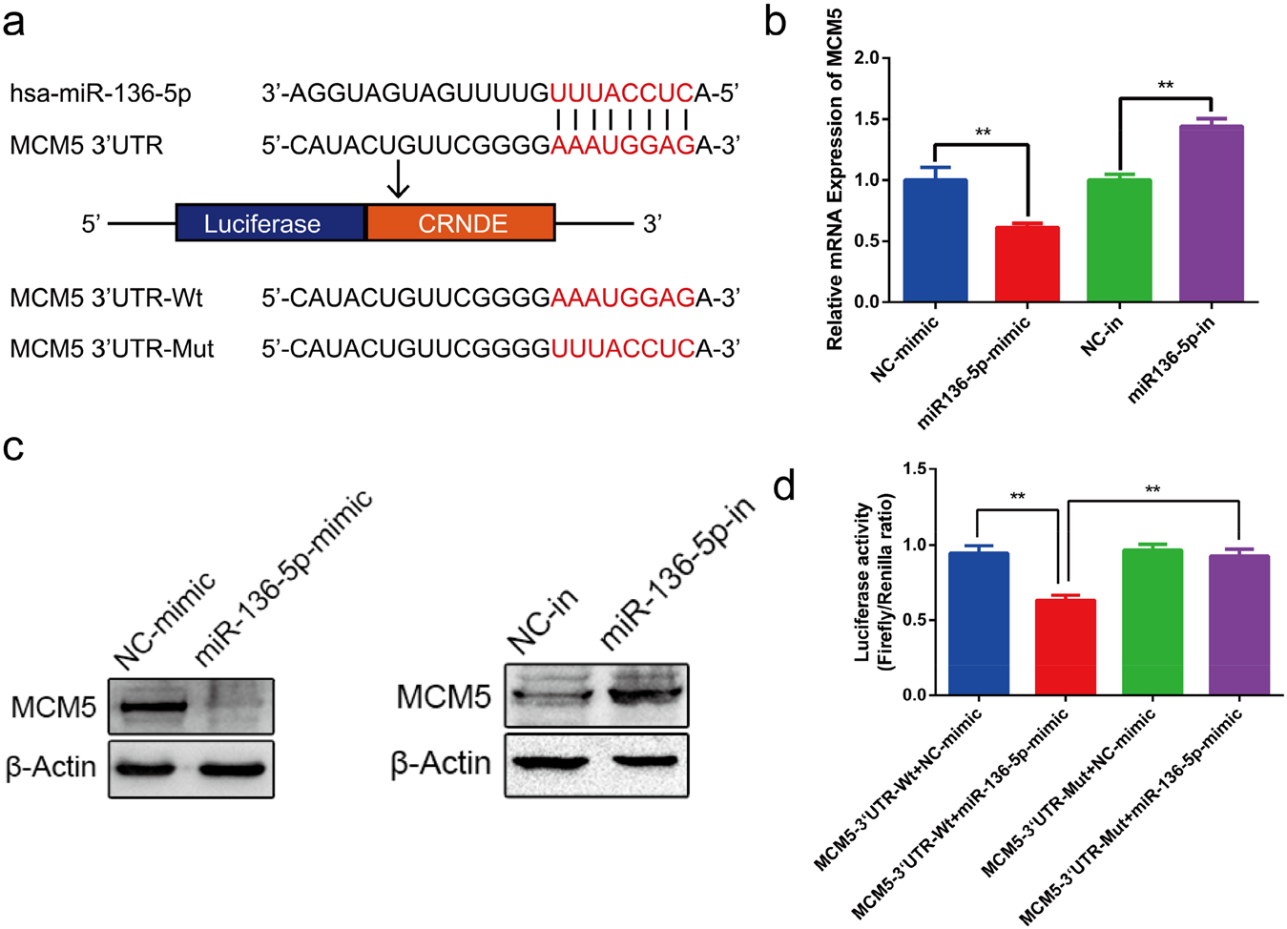
MiR-136-5p targets MCM5. (**a**) The binding sites between MCM5 and miR-136-5p were predicted by TargetScan 7.2 (http://www.targetscan.org/vert_72/). (**b**) QRT-PCR assay was used to detect the expression of MCM5 mRNA after transfecting mimic or inhibitor. (**c**) The expression of MCM5 protein was detected by western blot. 9**d**) Luciferase reporter assay for analysis of the interaction between miR-136-5p and MCM5. *p < 0.05, **p < 0.01.

To predict miR-136-5p direct target, bioinformatics software TargetScan 7.2 was applied and the results showed that MCM5 was a putative target of miR-136-5p. The binding sites between miR-136-5p and MCM5 are as shown in the Fig. 5a. The qRT-PCR results indicated that miR-136-5p inhibited MCM5 mRNA expression, while its expression increased following miR-136-5p down-regulation (Fig. 5b). These results were confirmed by Western blotting (Fig. 5c). Furthermore, the luciferase reporter assay displayed a decrease in luciferase activity in 293T cells cotransfected with miR-136-5p-mimics and MCM5-3’UTR-WT (Fig. 5d).

### CRNDE regulates MCM5 through sponging miR-136-5p

**Figure 6:**
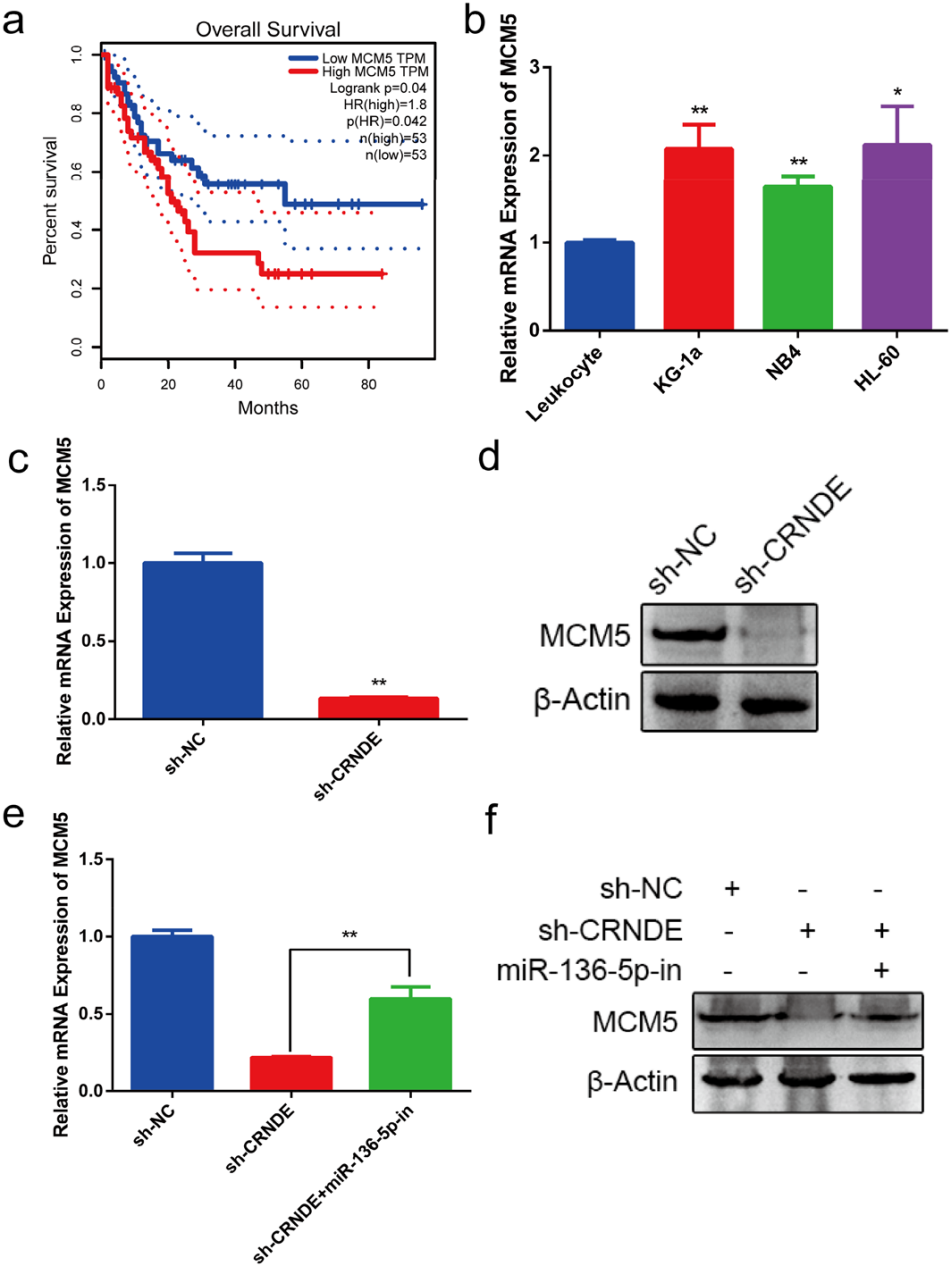
CRNDE regulates MCM5 through sponging miR-136-5p. (**a**) The overall survival curves of MCM5 in AML patients from GEPIA analysis. (**b**) MCM5 expression in AML cell lines was detected by qRT-PCR. (**c**) QRT-PCR assay was used to detect the expression of MCM5 mRNA after knocking down CRNDE. (**d**) Western blot was used to detect the expression of MCM5 protein after knocking down CRNDE. (**e**,**f**) Rescue assay was performed to verify that the effects could be reversed by miR-136-5p inhibitors at both mRNA and protein levels. *p < 0.05, **p < 0.01.

**Figure 7:**
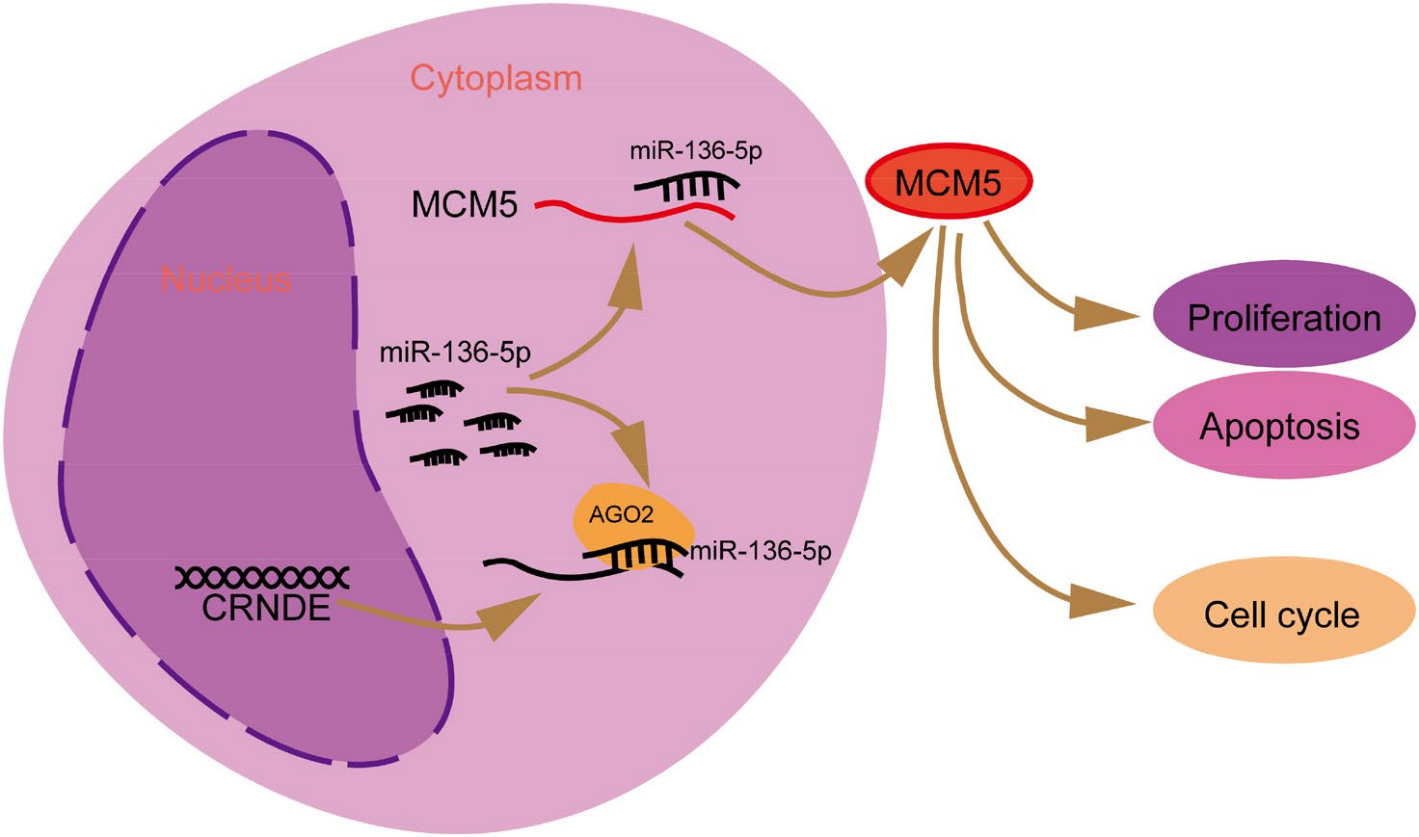
The possible modulating mechanism of CRNDE in KG-1a cells by sponging miR-136-5p.

To explore the effect of MCM5, we analyzed MCM5 expression data by GEPIA. The results revealed that high MCM5 expression correlates with poor overall survival in AML patients (Fig. 6a). Additionally, we also investigated the MCM5 expression in AML cell lines by qRT-PCR, and the results showed that its expression was highly expressed in AML cell lines (KG-1a, NB4 and HL60) compared with leukocytes (Fig. 6b). Moreover, the results of qRT-PCR and Western blot indicated that CRNDE knockdown inhibits MCM5 expression at the mRNA and protein levels (Fig. 6c,d). Rescue assays revealed that these effects could be reversed by miR-136-5p inhibitors, at both mRNA and protein levels (Fig. 6e,f), which further showed that CRNDE regulates MCM5 through sponging miR-136-5p. The possible modulating mechanism of CRNDE in KG-1a cells through miR-136-5p sponging is shown in Fig. 7.

## Discussion

AML occurrence and development are affected by multiple factors, such as radiation, chemical degradation, viral infection, and genes’ susceptibility^16^. Although a few chemotherapeutic drugs were applied in AML treatment, aged AML patients still suffer from poor prognosis and low long-term overall survival rate^17^. Increasing evidence revealed that lncRNAs play vital biological functions in tumorigenesis^18,19^. For example, Chen et al. revealed that CCAT1 acts as a molecular sponge of miR-181a-5p, regulates HOXA1 expression and promotes multiple myeloma progression^20^. Therefore, it is imperative to explore the potential mechanism of lncRNAs in hematological malignancies.

Emerging evidence revealed CRNDE usually promoted tumor progression. For example, Zheng et al. confirmed that CRNDE is the most upregulated lncRNA in glioma, where it promotes its progression by inhibiting the miR-384/PIWIL4/STAT3 axis^21^. Xu et al. revealed that CRNDE promotes melanoma migration and invasion via the miR-205/CCL18 axis^22^. CRNDE has been shown to be up-regulated in the bone marrow tissues of AML patients and negatively correlated patients’ overall survival^13^. However, the underlying mechanisms of it remain unexplored. In this study, we confirmed that the expression of CRNDE was significantly up-regulated in AML samples and cell lines. And CRNDE promoted KG-1a cells’ proliferation ability, decreases the apoptotic rate and arrested cells at the G1 phase. But, to our surprise, CRNDE high expression correlated with AML better overall survival (OS), which is contrary to existing conclusions. Considering that our results are based on a database and the sample size of the existing conclusions is small, we believe that it is necessary to bring clinical samples into subsequent experiments for verification.

The hypothesis of ceRNA was first presented by Salmena et al., who explained how messenger RNAs, transcribed pseudogenes, and long non-coding RNAs communicate with each other using microRNA response elements (MREs). They proposed that the “competing endogenous RNA” (ceRNA) could bind and negatively regulate the target^23^. Moreover, the function of lncRNAs was found to be closely related to its subcellular location^24^. In this study, lncLocator was used to predict the location of CRNDE^15^. We predicted that CRNDE was mainly located in the cytosol, where it potentially acts as a ceRNA. Subsequently, functional and mechanism experiments verified that CRNDE serves as a ceRNA for miR-136-5p and upregulates MCM5 expression by sponging miR-136-5p.

MiRNAs are a cluster of small single-stranded RNAs with a length of 18 to 25 nucleotides. Moreover, miRNAs participated in cell growth, cell apoptosis, hematopoietic lineage differentiation and cell death^25–27^. In our study, the results of the bioinformatics software Starbase V3.0 and LncBase V2.0 showed that miR-136-5p and miR-212-5p were downstream targets of CRNDE. However, the biological effects of miR-136-5p in AML have not been reported yet. Therefore, we take miR-136-5p as our first choice for further investigation. Our results showed that miR-136-5p is downregulated in AML cell lines and acts as a tumor suppressor. What’s more, the interaction between CRNDE and miR-136-5p was proved by luciferase reporter assays. Rescue assays also showed that CRNDE indeed influences MCM5 via acting on miR-136-5p.

Subsequently, we further explored the downstream target genes using Targetscan 7.2. In additional, Handschuh et al. listed the most overexpressed genes in AML through boutique microarrays, real-time PCR and droplet digital PCR^28^. So when you put these two things together, MCM5 was filtered out as a miR-136-5p direct target. Minichromosome maintenance protein 5 (MCM5) is a member of the minichromosome maintenance (MCM) protein family, that is located on chromosome 22q13.1^29^. MCM proteins were first identified in the yeast Saccharomyces cerevisiae as mutants defective in the maintenance of minichromosomes, indicating their role in plasmid replication and cell cycle progression^30^. MCM5 was well documented to play a role in tumorigenesis and cellular processes in various cancers. It has also been proven that MCM5 could always act as a prognostic marker in various cancers^31–36^, which makes it potential in clinic. In our study, we verified that MCM5 was upregulated in AML cell lines and associated with poor prognostic. Luciferase reporter assays showed MCM5 binds to miR-136-5p. And MCM5 was downregulated while CRNDE silencing or miR-136-5p overexpressing.

In the present study, we demonstrated the potential mechanistic role of CRNDE in KG-1a cells. And we also offered proof for the biological effects of miR-136-5p and the link between CRNDE and MCM5. However, there are still some limitations associated with the study. Firstly, we did not explore clinical samples, which is necessary to verify the effects of CRNDE on patient overall survival. Secondly, our research was performed in only one cell line, which was not abundant enough.

## Conclusion

In summary, we expounded that CRNDE plays a pro-oncogenic role in KG-1a cells, where it promoted proliferation by modulating the miR-136-5p/MCM5 axis. CRNDE-targeted therapy may be an effective strategy for the treatment of AML.

## Supplementary Information

Supplementary Information.

## Abbreviations

AML: Acute myeloid leukemia
CRNDE: Colorectal neoplasia differentially expressed
MCM5: Minichromosome maintenance protein 5
lncRNA: Long non-coding RNA
ceRNA: Competitive endogenous RNA
miRNAs: MicroRNAs
