# Supplementary material for: CRNDE enhances the expression of MCM5 and proliferation in acute myeloid leukemia KG-1a cells by sponging miR-136-5p
Source: Sci Rep. 2021 Aug 18;11:16755. doi: 10.1038/s41598-021-96156-3 (PMC8373925; doi:10.1038/s41598-021-96156-3)
Supplement: Supplementary file 1 — Supplementary Information. [file 41598_2021_96156_MOESM1_ESM.pdf]

CRNDE enhances the expression of MCM5 and proliferation in acute myeloid leukemia KG-1a cells by sponging miR-136-5p

Chen Liu[1, 2], Liang Zhong[2], Chenlan Shen[1], Xuan Chu[1], Xu Luo[1], Lihua Yu[3], Jiao Ye[2], Ling Xiong[1], Wenran Dan[1], Jian Li[2] and Beizhong Liu[1, 2]

Fig.S1a Genes predicted by Starbase V3.0.

hsa-miR-4424	hsa-miR-337-3p	hsa-miR-556-3p
hsa-miR-6835-3p	hsa-miR-3144-3p	hsa-miR-338-3p
hsa-miR-29a-3p	hsa-miR-212-5p	hsa-miR-323b-3p
hsa-miR-29c-3p	hsa-miR-1277-5p	hsa-miR-136-5p
hsa-miR-29b-3p	hsa-miR-545-5p	hsa-miR-384

Fig.S1b Genes predicted by LncBase V2.0.

hsa-miR-7856-5p	hsa-miR-212-5p	hsa-miR-4712-5p
hsa-miR-641	hsa-miR-212-5p	hsa-miR-3157-5p
hsa-miR-641	hsa-miR-153-5p	hsa-miR-543
hsa-miR-641	hsa-miR-153-5p	hsa-miR-543
hsa-miR-6787-3p	hsa-miR-4712-5p	hsa-miR-3157-5p
hsa-miR-641	hsa-miR-136-5p	hsa-miR-4712-5p
hsa-miR-6847-5p	hsa-miR-212-5p	hsa-miR-543
hsa-miR-5700	hsa-miR-205-5p	hsa-miR-4680-3p
hsa-miR-335-3p	hsa-miR-4680-3p	hsa-miR-3157-5p
hsa-miR-335-3p	hsa-miR-4712-5p	hsa-miR-1273h-3p
hsa-miR-335-3p	hsa-miR-4680-3p	hsa-miR-3157-5p
hsa-miR-153-5p	hsa-miR-4282	hsa-miR-6768-3p
hsa-miR-212-5p	hsa-miR-4712-5p	hsa-miR-4680-3p
hsa-miR-335-3p	hsa-miR-1273h-3p	hsa-miR-1250-3p

Fig.S2 Genes predicted by Targetscan 7.2.

AAK1	C7orf73	DRAM1	GRIK1-AS2	MOB1B	PCBD2	RIMKLB	TFAM	ZKSCAN4
ABCC12	CACNA2D2	E2F1	GTF2I	MON2	PCDH1	RLIM	TFCP2	ZNF24
ABHD14B	CALCOCO2	EFHC1	HAL	MORC1	PCTP	RNF121	TLL1	ZNF32
AC006486.1	CALCR	EIF3A	HHIP	MOSPD1	PDK3	RNF214	TM9SF3	ZNF451
ACAP2	CAND1	ELF3	HIPK2	MPZL1	PEG10	ROCK1	TMEM196	ZNF462
ACSF2	CASP8	ELFN2	HOOK3	MRPS14	PFAS	RPGRIP1L	TMEM38B	ZNF471
ADAM23	CBL	ENAH	HOXC10	MRVI1	PGBD5	RPRD1A	TMEM9	ZNF618
ADAT2	CBX4	ENTPD1	HOXD11	MSL2	PHEX	RPUSD4	TMPRSS11F	ZNF638
ADCYAP1R1	CCDC171	ENTPD7	HS3ST1	MTDH	PHF20	RRP15	TNPO1	ZNF710
ADRBK2	CCDC180	EPG5	HSD17B10	MTMR4	PHF21A	RYBP	TNPO2	ZNF827
AGAP2	CCDC58	ERI2	HSPA13	MTPN	PHKA1	SAR1B	TNRC18	ZRSR1
AGO1	CCER1	ESRRG	HYPK	MYNN	PIP5KL1	SEMA4C	TOR1AIP2	
AKIRIN1	CCPG1	ETF1	IER3IP1	MYPN	PLCXD3	SEPT7	TP53RK	
AL021546.6	CD163	ETV5	INO80	NAALADL2	PLD5	SETD8	TPRG1	
ALPK3	CD3G	EXOSC2	KATNAL2	NABP1	PLEKHO1	SGIP1	TRIM27	
ALS2	CDH7	EXPH5	KCNJ13	NANOS1	PLXNB1	SH2B1	TRIM52	
ANKAR	CDK12	EXTL3	KCNK10	NARF	POMGNT1	SHISA9	TRPC4AP	
ANKRD11	CDV3	FAF1	KCTD16	NCOA2	PPARGC1A	SLC2A8	TSC22D2	
ANKRD63	CLPP	FAM120A	KDM3A	NF2	PPARGC1B	SLC7A14	TSPAN3	
AP3M1	CMTR1	FAM229B	KIAA1522	NFIB	PPP1R18	SLC7A3	TSPAN33	
ARHGEF15	CNDP1	FAM63B	KTI12	NOTCH3	PPP2R2A	SMG1	UBE2N	
ARHGEF4	CNIH4	FAM84B	L3MBTL1	NOVA2	PPTC7	SNCB	UBE2R2	
ARID5B	CNOT7	FAT3	LIFR	NPAS3	PRDM16	SOCS7	UBTD2	
ARL5B	COG5	FHDC1	LIN7A	NPFFR1	PRDM6	SORCS1	UQCC1	
ATXN1L	COL6A6	FHL5	LMO4	NR2F2	PRICKLE1	SP2	USP37	
AZIN1	COPS4	FOXN2	LRIG3	NR4A3	PRPF4	SP8	USP4	
B4GALT2	CPD	FRAT1	LRRC55	NRK	PSD3	SRSF1	VCAN	
BCL9L	CPEB2	FRK	LUZP6	NUBPL	PSME4	SRSF9	VCP	
BMPR1A	CPEB4	FZD4	MAP2K4	NYNRIN	PTPN3	SS18L2	VMAC	
BMPR2	CREBZF	GALNTL6	MAPK1	OCIAD1	PURB	STIM2	WASF3	
BNC2	CREG2	GANAB	MAT2B	OIP5	PWWP2A	STRIP2	WDR43	
BPTF	CYB5RL	GATC	MATN1	OSBPL3	RAB9A	STRN3	WNT2	
BTLA	CYP3A5	GCC1	MCM5	OTUD3	RABGAP1L	STT3B	XIAP	
BZW1	DCAF7	GDF6	MEI4	OXLD1	RABGGTA	SUDS3	XPO7	
C10orf118	DCP2	GDI2	METTL1	P2RY2	RABL3	SUGT1	YARS2	
C10orf12	DGKI	GDPD5	MEX3C	PAOX	RAD23B	SUV39H1	ZBTB20	
C10orf2	DIO2	GINS1	MGAT5	PAQR3	RAP2C	SV2B	ZBTB21	
C17orf47	DIS3	GLIPR2	MGRN1	PARVA	RBMS2	SYDE1	ZBTB5	
C18orf32	DLG3	GNAS	MID2	PATL1	RDX	TAL1	ZBTB6	
C1QTNF7	DMXL1	GNG3	MIDN	PAX5	RET	TANC2	ZC3H10	
C21orf119	DNMT3A	GPC6	MIEN1	PAX6	RGS4	TBCEL	ZCCHC14	
C5orf24	DOCK11	GPR26	MINK1	PBX1	RGS7BP	TCEANC2	ZDHHC6	
C6orf62	DOCK5	GRID1	MLLT11	PBX3	RIMKLA	TET3	ZFYVE28	

Fig. 2

e Cell apoptosis was detected by western blot. **g** Cell cycle was detected by western blot.

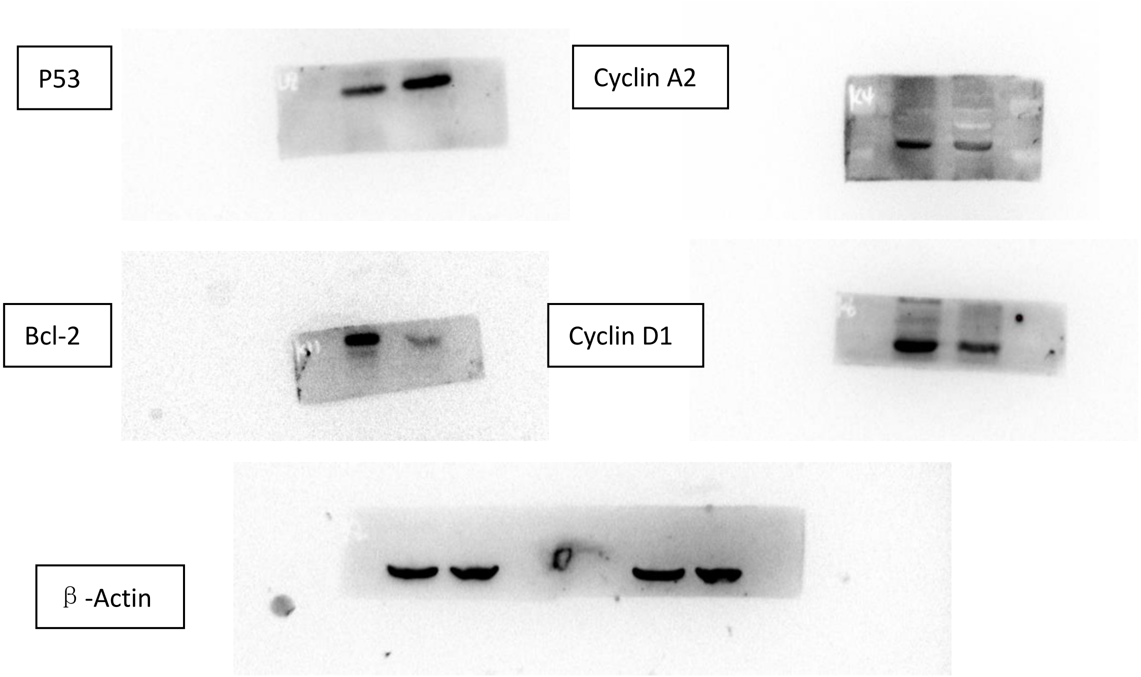

Fig. 5

c The expression of MCM5 protein was detected by western blot after translating mimics or inhibitors.

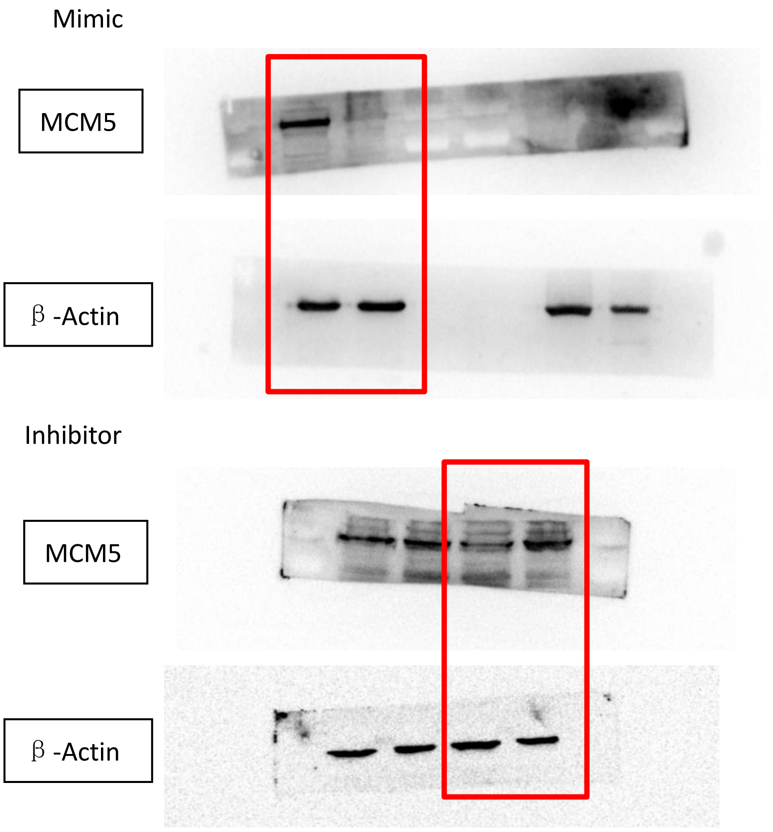

Fig. 6

d Western blot was used to detect the expression of MCM5 protein after knocking down CRNDE.
f Rescue assay was performed to verify that the effects could be reversed by miR-136-5p inhibitors at protein levels.

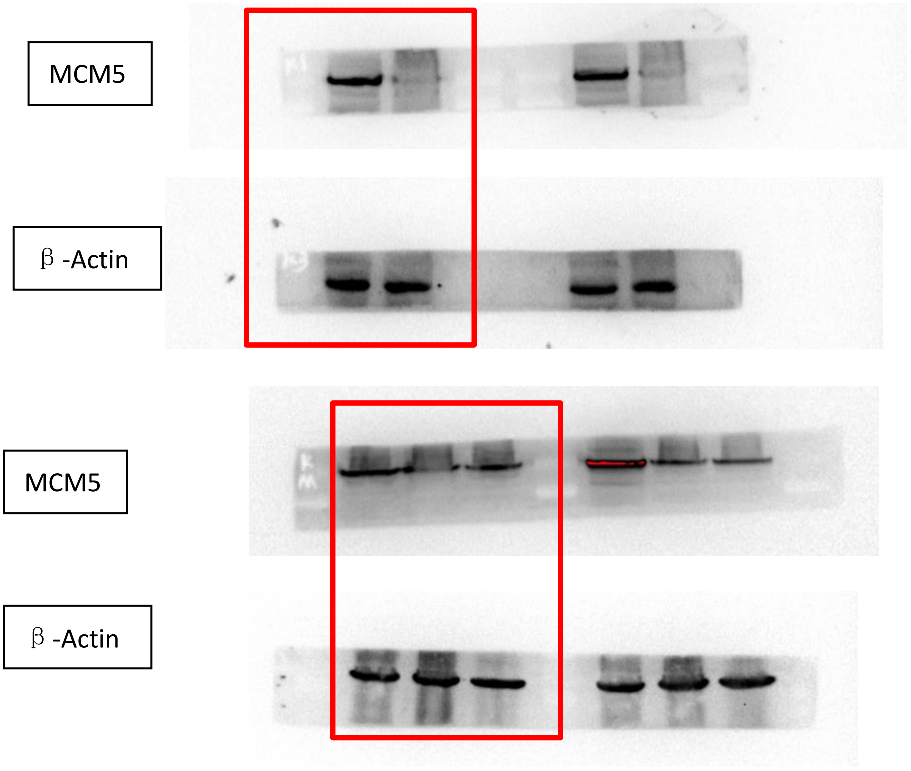

Ethics approval and consent to participate

All experimental protocols were approved by Chongqing Medical University. Protocols involving human research participants had been performed in accordance with the Declaration of Helsinki and informed consent was obtained from all subjects.

The Ethics Committee of

ChongQing Medical University

Approval Notice of research paper

Principal Investigators: Chen Liu

Title of Projects: LncRNA CRNDE Promotes AML Progression via Sponging MiR-136-5p.

Date Submitted: 2021.3.1

Date Reviewed: 2021 . 3 . 12

Date Approved: 2021 . 3 . 18

The Ethics Committee of Chongqing Medical University has reviewed the proposed use of human subjects in the above mentioned projects. It is recognized that the rights and the welfare of the subjects are adequately protected; the potential risks are outweighed by potential benefits. We approve papers resulting from the project.

The Ethics Committee of Chongqing Medical University
